# An Old-Time Syphilidiater

**Published:** 1910-12

**Authors:** Hubert J. Norman

**Affiliations:** Assistant Medical Officer, Camberwell House Asylum, London.


### AN OLD-TIME SYPHILIDIATER.

BY

HUBERT J. NORMAN, M.B., D.P.H.

*Assistant Medical Officer, Camberwell House Asylum, London.*

AMONG the many and varied diseases which practitioners of the therapeutical art have had placed before them by outraged nature, it is doubtful if any condition in pathology has occupied a more prominent position than the one which has received the name of the "Venereal Disease." Its history passes back to the most dim obscurity, and is finally lost in the mists of time ; and, while many lands would gladly claim to have been the birthplace of the author of the Homeric epic, there is a striking disinclination on the part of any country to arrogate to itself the distinction of having been the primal focus from whence this condition has spread to the uttermost confines of the earth. It is probable that the question of its origin will never be solved, for the complications which hinder a successful solution are numerous. As is well known, many theories have been advanced, each of which has in its turn been considered satisfactory by a certain proportion of the human race, in which latter category it has seldom been possible to include the most favoured nation to whom the charter of rights and precedence has been willingly granted by their neighbours. It may be true that Columbus and his sailors when they discovered America also discovered, unwittingly we may be sure, venereal disease. On the other hand, it is possible that what one of his expedition claimed as a novelty may simply have been an encroachment upon the property of one of his comrades. And so with Captain Cook and his company and with many others, even to the Crusaders and earlier than their days; for enactments were enforced in regard to "stews" and the "brenning" at a very early date.

Such discussions as these are, however interesting, not the subject of the present essay, which is rather concerning the therapeutics of venereal disease, as exemplified in an account by a writer of the period, in the early part of the eighteenth century; a time when, if accounts and diagnosis may be believed, such disorders were not only extremely prevalent, but were also a common topic of conversation, formed the subject of mild reproaches, and were on occasions matter of highly-specialised objurgation. Smollett, who seldom erred on the side of obscurity in such matters, and who was besides a medical man, will furnish a criterion.

The dogmatist, in whatsoever profession or trade he may be found, is useful in some degree, though when he is, for instance, the discoverer of a cancer "cure" his utility is not always on the surface. Mr. John Profily, M.D., who wrote early in the eighteenth century his work on venereal disease, suffered under no delusion as to his capabilities for dealing therapeutically with the protean disease, which was even more protean then than it is now, if we may judge from his comprehensive symptomatology. He calls his book *An Easy and Exact Method of Curing the Venereal Disease.* It is interesting to note the dogmatism of his " curing " when it is noted that he blends in one indistinguishable whole syphilis, gonorrhœa, and soft sore. John Hunter had not yet discriminated for him between the chancre, which so tragically bears that great investigator's name, and the soft variety ; therefore Dr. Profily was enabled to effect cures of the venereal disease which were so numerous that he might have been even prouder had they all been actual rehabilitations of bodies undoubtedly infected with the syphilitic virus. He continues his title, *With an Account of its Nature, Causes, and Symptoms: Demonstrated by way of Dialogue between Physician and Patient; for the Use and Instruction of all unfortunate Persons who may labour under that Disorder: by which in common* (it would be interesting to know more explicity his interpretation of this term) *Cases they may be instructed to cure themselves, or to know if they are treated according to Art by those whom they apply to.* Not content with so ambitious a scheme, he adds, *A Method of curing the Scurvy, Gleets, Whites, etc.*, and further subjoins, *Experiments publickly made on several Patients of an effectual and safe Method of curing the said Disease, without Salivation or Confinement.* Note in passing the necessity which leads him to make mention of the fact of cure without salivation, an interpolation which gives much information as to what was the usual procedure in his day. After a dedication addressed, " Clarissimis Dominis Praesidi et Sociis Collegii Medicorum Londinensis," he proceeds to disclaim in his preface any intention of writing " With a private View of Lucre and Profit," and addresses some scathing remarks to the " Presumptuous Pretenders," or quacks of his day, " who arrogate to themselves the sole power of curing this Disorder." Nevertheless, in spite of this fine altruistic spirit, which leads him here and in other places to deny his desire of lucrative return for his labours, we find him vaunting the powers of his "Greek-water and Pills" in the cure of venereal troubles; and a page preceding the first dialogue is given up to an advertisement which gives the information to all whom it may concern that "Greek-water or Antiphrodisiac Pills may be had only at Mr. John Cartwright's."

We then come to the dialogues proper. The thirst for accurate scientific information in patients, which leads them to expect a detailed explanation of the rise, progress and terminations of their diseases, which is considered by many as the outcome of more extended knowledge in the present day, and which is promoted by the more or less accurate monographs appearing in the encyclopædias, is of no such recent growth, if Dr. Profily's patients may be taken as an index of the characteristics of the time. They ask for details, etiological, pathological, anatomical, therapeutical, and they are given full measure, heaped up and brimming over. He gives them his ideas of the causation of their trouble, he details the symptoms, he dilates on their anatomical structure, not merely of their "privities," but introduces a minute description of the composition of the eye and ear, and illustrates them with imposing folding copper-plates. Perhaps the most astounding fact is that after a long, learned (not to say verbose), disquisition, the patient will affirm with a certainty which does great credit to his imagination that he has understood fully what gives pause to the modern reader. If, however, in this regard the patient of those days differs in intelligence from at least the average patient of the present day, there is yet a striking similarity in the rise of his troubles. "I chanced to pass the evening with some friends at the Tavern, and getting into liquor, was persuaded by one of the company (who in that instance was little a Friend to me or to himself) to adjourn to an House, it would be prudent for a man never to set his Foot into. I was too much intoxicated to have any memory of what happened there; but from the Sequel I conclude, with more than Conjecture, that I must have had carnal Commerce with an infected Female." There is a fine biblical rhetoric in the statement of another patient, who varies periphrasis to plain bluntness in an admirable manner. "Friend," he addresses the physician, "two Years ago led by a wicked Spirit of the night, in whom there was no Truth, I had an affair with one of the Race of Ungodliness who gave me what thy People calleth a Clap. I ran in full fervour of Pain to what thou willst call a Quack, (for he had not been Friend, to what thou callest Universities), he gave me an Electuary which stopt a dismal Running I had, and I went home rejoicing and returning Thanks to the Lord that he had thus delivered his Servant from the Hand of Vengeance." His thanks seem, however, to have been somewhat premature, and are not chronicled when "in three month's Time two Ulcers appeared on the Top of the lustful Member, and one of the round Supports began to swell." So he underwent a salivation, which "sent away the Marks of Satan from my Body." But his trouble with his "Unruly member," as he elsewhere calls it, does not cease here, for the learned physician informs him that should "the Disorder spread to the spermatick Rope, and from thence to the Cavity of the Abdomen, then the Testicles must be cut off for fear of their degenerating into an Abscess or becoming cancerous." Whereupon the miserable man exclaims: "Oh, how desolate and afflicted is thy Servant, O Lord, for a transitory effort; and how deceitful that Piece of Flesh that I had to deal with, even the Flesh of Rebecca. But, my good Friend, I can assure thee that I am Not willing to take away these Instruments of Desire till I have made use of all that thy Business can help me in."

Let us, however, pass over all his long disquisitions upon such subjects as the origin of the fluid in a gonorrhœa, its yellow colour, the structure of the ear and of the eye, the fluor albus, scurvy, and so on, except to note that when dealing with the ear he recommends that the meatus auditorious may be kept clean by washing it with urine, and says that the "most inveterate Deafness may be cured by means of the Eggs of Ants bruised and put into the Ear with the Juice of an Onion": and let us see what are the lines of treatment he follows, primarly in regard to prophylaxis. A patient having suggested that it might be well that he should hear the physician's "Opinion of a Preservative against the Disorder," is answered, "The most certain Preservative against this Disorder is to live chastly and to avoid Incontinence." Whereupon the patient pertinently remarks, "Sir, I don't consult you as a Moralist but as a Physician." Follows an interlude anent the ethics of prophylaxis, during which the physician, mingling morality with economy, says, "Such a Preservative would, in the opinion of some, be thought an Encouragement to Vice, and hurt many others who, in a great measure, depend on the Cure of this Disorder for their Maintenance." He quotes Fallopius(*De Morbo Gallico*) on the point, whose opinion is that "there are ways of preventing Ulcers arising from Contagion," and he recommends that the penis should be wiped with linen after coitus, the linen having been prepared for action by being steeped in a solution which contains some fourteen ingredients. "In hoc decocto maceretur per noctem pannus linteus purissimus." Further, Fallopius tried this in 1,100 cases, not one of whom was infected, whilst other writers got almost as good results with simple washing and cleanliness. Then there is a consideration of the condom—cundum he calls it—a term more familiar in the writings of American authors nowadays than works written in this country. *The Lexicon of Medicine* states that the origin of the term was in the name of a London physician, called Conton, who lived in the eighteenth century, and first suggested its use. This preventive, epigrammatically described as a "Cobweb against danger, a Cuirass against pleasure," was made, according to Profily, of "lamb's bladder." He quotes Astruc's curious objection to its use: "We 'll suppose," Dr. Astruc says, "the Bladder don't break, but remains entire through all the Act, still all is not safe, neither the Pubes or the Groin are covered; those Parts therefore remain still obnoxious to the spermatick Humour."

In regard to the treatment of the disease after it has actually arisen he speaks with no uncertain voice. The following quotation will give a succinct idea of his procedure, and will at the same time exemplify his confusion of the varieties of venereal diseases, a confusion common in his time. "The common practice of curing a Gonorrhea is to give Mercur. Sublimat. Dulc., mixed with some purgative medicine, or to give it alone at night, and purge it off the next morning; and then if the Running continues, to give some healing Medicines, and use an Injection to complete the Cure. My new Method is to give, instead of Mercur. Sublimat. Dulc., some Crude Mercury, commonly called Quicksilver, in purging Pills, and then some astringent Medicines, and my Greek-water by injection." He is a firm believer in the value of drinking large quantities of bland fluids; he prescribes barley-water and marsh-mallow as a draught, several pints of this compound to be drunk morning and evening, or a quart of gruel at a time, or of "thin light broth." The patient must not eat "salt or high-seasoned Meat," but anything else evidently that he pleases: or he may drink "common Table-beer, Wine, and Water, or any other innocent liquor," but must refrain "as far as possible" from intemperate drinking. A point to be noted is that he advises that the injection be used after the patient has made water, a matter too often overlooked even at the present time. Though some of his prescriptions include ten or more ingredients on occasions, he seems to place little faith in internal medication, and relies more upon the injections: the medicine which he gives is more of the nature of a placebo, and more for its mental effect. This leads him to a digression on syphilophobia, and he adduces the opinion of the "learned Dr. Friend" (more correctly "Freind"), that "an honest Practitioner generally finds it more difficult to cure the imaginary Evil than real one," a statement which has lost very little of its applicability even in the present day.

In regard to epididymitis and its cure there is a curious instance given of what the charlatans of those days considered rational treatment; interesting too in this, that even now, when knowledge seems to be widespread, among certain classes connection with a virgin is looked upon as the most efficient treatment of gonorrhœa. It is difficult to understand how such an idea can have arisen, while the morality of those who suggest and of those who follow such a line of treatment is simply despicable. The patient has seen an advertisement in a newspaper of remedies for the "Cure of almost all Disorders," and has—following the usual custom of the tribe—sent his money in advance. "I was surprised when I saw the Letter which he wrote me, wherein he sent me what he calls a Practical Scheme, and prescribed me to lie with a Woman, if I could, to clean the Spermatick Vessels; and tho' Venery, says he in this Letter, otherwise in the Disease hinders the Cure, yet in your Case would much contribute to cure swell'd Testicles." Both Profily and his patient strongly deprecate such advice, it is gratifying to observe, and the physician recommends a bolus containing cassia, rhubarb, and calomel; a plaster containing, among other ingredients, lupine, fenugreek, rye, and cumin, which is to be applied warm to the part, and the scrotum to be supported by a truss.

Syphilis as an entity, as we have seen, he did not discriminate; but when ulcers appeared on what, in quite Chaucerian phrase they called the "Yard," there were various forms of treatment of a more stringent character; when, too, there were rashes on the body, pains in the throat and head, it was seen that the trouble was more deep-seated. It may be said, in a word, that mercury is some form was the basis of all treatment. There was fumigation, which was directed against the disease when it appeared in the nose or throat, and was carried out by enclosing the patient in a blanket and then throwing a drachm of finely-powdered cinnabar on to a heater which reposed on an earthen platter held on the knees. Interesting in connection with the modern revival of the sarsaparilla treatment by patent-medicine vendors, and even by the medical profession (as in Zittmann's decoction), is the fact that a ptisan or decoction of sarsaparilla and guaiacum is recommended. Inunction was advised and carried out to a degree which would cause alarm now. Unless salivation were so profuse that the expectoration amounted to five or six pints a day it was not considered that a sufficient amount of mercury was being given, and the patient's mouth had to be ulcerated—" for the spitting depends on that"—his teeth loose, his tongue swollen, his mouth foul, his breath fœtid, and his gums spongy and sore. Little wonder that some preferred their "pox." His speciality, however, was his treatment by mercurial pills, and by these he obtained good temporary results, but when the immediate symptoms had disappeared treatment was discontinued. The natural result was that tertiary symptoms followed at a later date. They were not, however, allowed in any way to detract from the credit of the physician who had effected a "cure" in the primary or in the secondary period. Their full significance was not clearly understood, though the same might with no small amount of justice be said at the present day.

It were too lengthy a task to follow the good doctor into all the minutiæ of his treatment, and would, indeed, serve no useful purpose. A few words may, however, be said of the etiological ideas of those times. They seem to foreshadow in a curious way modern discoveries, including even that of the spirochætes. "I will refer you," he says to one inquisitive patient, "to the sentiments of Abercromby, Deidier, Desault, and others, who are of opinion that the Venereal leaven consists of imperceptible Worms, which, on approaching, are communicated from one Body to another, and afterwards multiply in the Person who has received them." These organisms he designates, in a phrase reminiscent of the delightful Carrollean, "Slithy Toves,"—"Pocky Worms."

So with this prevision let us take leave of this early coadjutor of the messenger-god in his combats against the afflicted of the fairest of the goddesses, and let us hope that as his work helped the knowledge of his time forward, so this brief epitome will add both to the information and to the entertainment of this later day.

